# Elastofibroma Dorsi Detected Incidentally on Chest Computed Tomography: The Prevalence and Reporting Rate in Radiology Reports

**DOI:** 10.7759/cureus.51280

**Published:** 2023-12-29

**Authors:** Gurbet Yanarateş, Nurdan Fidan

**Affiliations:** 1 Radiology, Hitit University, Erol Olçok Training and Research Hospital, Çorum, TUR; 2 Radiology, Hitit University Faculty of Medicine, Çorum, TUR

**Keywords:** elastofibroma dorsi, computed tomography, prevelance, radiology reports, density, thickness

## Abstract

Background

Elastofibroma dorsi (ED) is an uncommon benign connective-tissue tumor, usually seen in the subscapular region of women after the fifth decade. The study aimed to determine the prevalence, radiological characteristics, and the rates of mention in the initial radiology reports of ED incidentally detected by chest computed tomography (CT) imaging in a large population.

Methodology

This study was conducted at Hitit University, Erol Olçok Training and Research Hospital Radiology Department in Çorum, Turkey. A total of 3,299 patients (1,554 females, 1,745 males) who underwent non-contrast chest CT for various reasons were included in this retrospective study. The presence, side, thickness, and density of ED were investigated in these patients. Differences in gender and laterality were assessed statistically. It was also reviewed whether it was stated in the initial radiology report or not.

Results

ED was detected in 79 (2.4%) of 3,299 patients, in 60 (75.9%) females and 19 (24.1%) males with a median age of 57.5 years (range, 18-99 years). The risk of ED presence was determined to be 3.65-fold in females than in males. In the cases determined with ED, the median age was 75 years (range, 53-96 years), and ED was not determined in any patient aged <50 years. A statistically significant correlation was determined between age and the presence of ED (p < 0.001). No statistically significant correlation was found between age and ED thickness or density (p = 0.602, p = 0.233, respectively). It was noted that none of the patients were diagnosed in the first radiological report.

Conclusions

ED can be easily overlooked on cross-sectional examinations because of the similar appearance and density to adjacent structures. Knowledge of the characteristic radiological features of these lesions and increased awareness of radiologists will make early diagnosis possible in asymptomatic cases.

## Introduction

Elastofibroma dorsi (ED) is a slow-growing, benign soft tissue mass of mesenchymal origin. It is a fibroblastic/myofibroblastic tumor formed from thick elastic fibers within a lipid-rich collagen matrix [1]. In 99% of cases, it is localized between the scapula and thoracic wall, between the rhomboid major, latissimus dorsi, and serratus anterior muscles in the inferior subscapular region [2]. This localization has been reported as pathognomonic for ED [3]. Other less common localizations are the lateral chest wall, deltoid muscle, axilla, trochanter major, olecranon, foot, tricuspid valve, tuberositas ischii, inguinal region, omentum majus, stomach, rectum, spinal canal, sclera, orbit, and the mediastinum [4]. 

Periscapular localisation is usually seen in females in the fourth to seventh decades of life. In patients aged >60 years undergoing thoracic computed tomography (CT), the prevalence has been reported as 2%, but in autopsy studies, it has been seen at a higher rate (13-17%) [5]. Although the majority of localizations are unilateral and on the right side, bilateral involvement has been reported at rates of 10% to 66%.

The etiology is not fully known, but it has been suggested that it is a reactive pseudotumor related to mechanical friction between the scapula and costae. This view is supported by the fact that localization in most cases is in the right periscapular region and the dominant hand is right side. However, lesions in different localizations and seen in those who are not engaged in heavy manual labor show that hereditary and structural factors are effective in the growth of the lesion [6].

There are various studies in literature related to the imaging findings and prevalence of ED in radiological and autopsy series, but a limited number of studies have been conducted on extensive populations for prevalence and demographic data. Moreover, although the imaging findings and typical localization are known, ED can be overlooked or misdiagnosed on routine thoracic CT examinations. The aim of this study was to determine the prevalence, radiological characteristics, and the rates of mention in initial radiology reports of ED detected incidentally on chest CT imaging in a large population.

## Materials and methods

This retrospective study was checked for compliance with the Helsinki Declaration of Human Rights and was approved by Hitit University, Erol Olçok Training and Research Hospital's ethics committee before the study (date: March 31, 2022; decision number: 2022-25).

The study included consecutive adult patients who had chest CT scans taken for various pre-diagnoses in in Hitit University, Erol Olçok Training and Research Hospital Radiology Department between January 2021 and January 2022. Patients were excluded from the study if they were aged <18 years, if history of trauma or an advanced degree of edema was detected on the CT scan, if image quality was poor, or if there was invasion to the thoracic wall.

All the images were taken with the patient positioned supine, using a 128-slice multi-detector CT scanner (Optima 660, GE MedicalSystem, Milwaukee, WI, USA), and axial slices were acquired without IV contrast material administration. The CT scan protocol was as follows: slice collimation = 320×0.5 mm, gantry rotation time = 0.275 s, tube voltage = 120 kV, and automatic tube current = 120-380 mA. The CT images were retrospectively examined by a single radiologist with at least five years experience in this field, and when necessary, the findings were confirmed by a radiologist with 10 years of experience. The presence of ED on the CT scans was evaluated according to typical diagnostic findings, such as typical localization of the lesion in bilateral periscapular regions and density similar to that of the adjacent muscle.

For each patient, a record was made of demographic data, such as age and gender, the presence of an ED lesion, the side, the thickness of the lesion in the axial plane, and density. The initial radiology reports on the day when the CT was taken were also reviewed to determine whether or not an ED lesion was reported.

Statistical analysis 

Data obtained in the study were analyzed statistically using IBM SPSS Statistics for Windows, version 22 (released 2013; IBM Corp., Armonk, New York, United States). Conformity of the data to normal distribution was assessed with the Shapiro-Wilk test. Descriptive statistics were stated as mean±standard deviation (SD) or median values for continuous variables and as number (n) and percentage (%) for categorical variables. In the comparison of numerical data showing a normal distribution, the Student’s t-test was used, and for the comparison of categorical data, the Pearson Chi-square test was applied. A value of p < 0.05 was accepted as statistically significant.

## Results

Evaluation was made of the CT scans of 3,299 patients, including 1,554 (47.1%) females and 1,745 (52.9%) males, with a mean age of 57.5 years (range, 18-99 years). ED was detected in 79 (2.4%) of 3,299 patients; 60 (1.8%) were females and 19 (0.6%) were males (Table 1).

**Table 1:** Descriptive statistics. ED: elastofibroma dorsi, HU: Hounsfield unit

Total patients (n)	3299
Age (years)	
Total (mean,range)	57.5 (18-99)
ED (+) (median,range)	75 (53-96)
Gender, n (%)	
Male	1745 (52.9%)
Female	1554 (47.1%)
Presence of ED, n (%)	
Male	19 (0.6%)
Female	60 (1.8%)
Total	79 (2.4%)
Distribution of ED, n (%)	
Right	20 (25.3%)
Left	12 (15.2%)
Bilateral	47 (59.5%)
ED thickness (mm)	
Right	13.47 (7-25)
Left	11.87 (5-25)
Total	12.69 (5-25)
ED density (HU)	
Right	+20 (-41 to +70)
Left	+21 (-46 to +90)
Total	+24 (-46 to +90)

**Figure 1:**
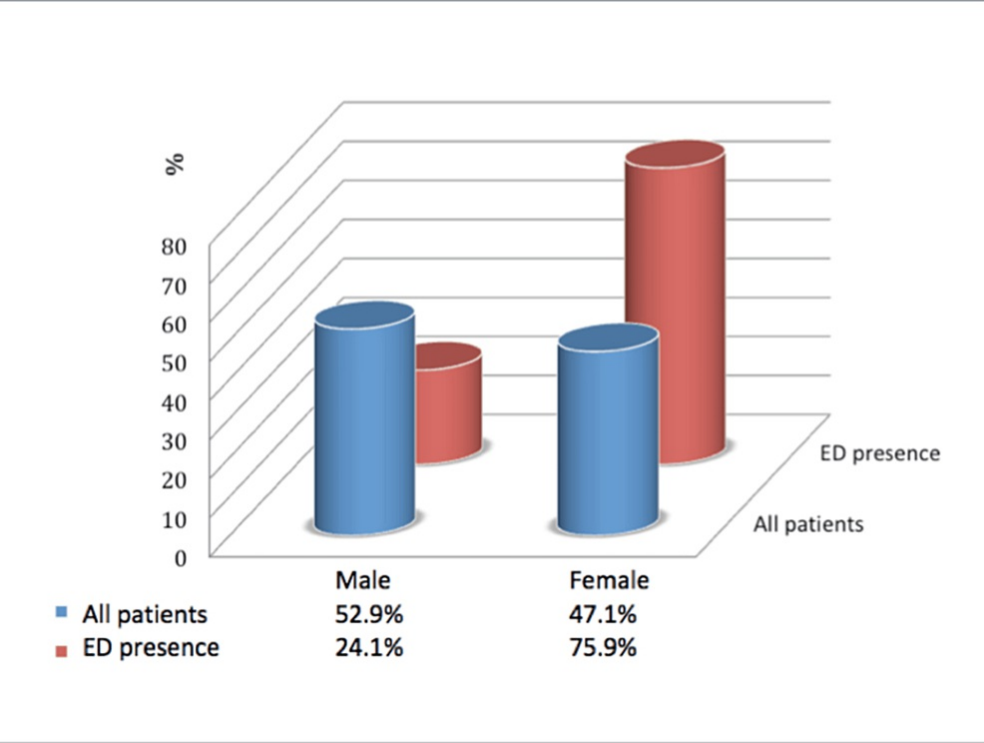
Gender distribution in all patients and the presence of ED

The ED-detected patients were 75.9% female and 24.1% male (Figure 1). ED was significantly more common in female patients than in male patients (p < 0.001). The risk of ED presence was determined to be 3.65-fold greater (confidence interval (CI) 95%, 2.17-6.13) in females than in males.

**Figure 2:**
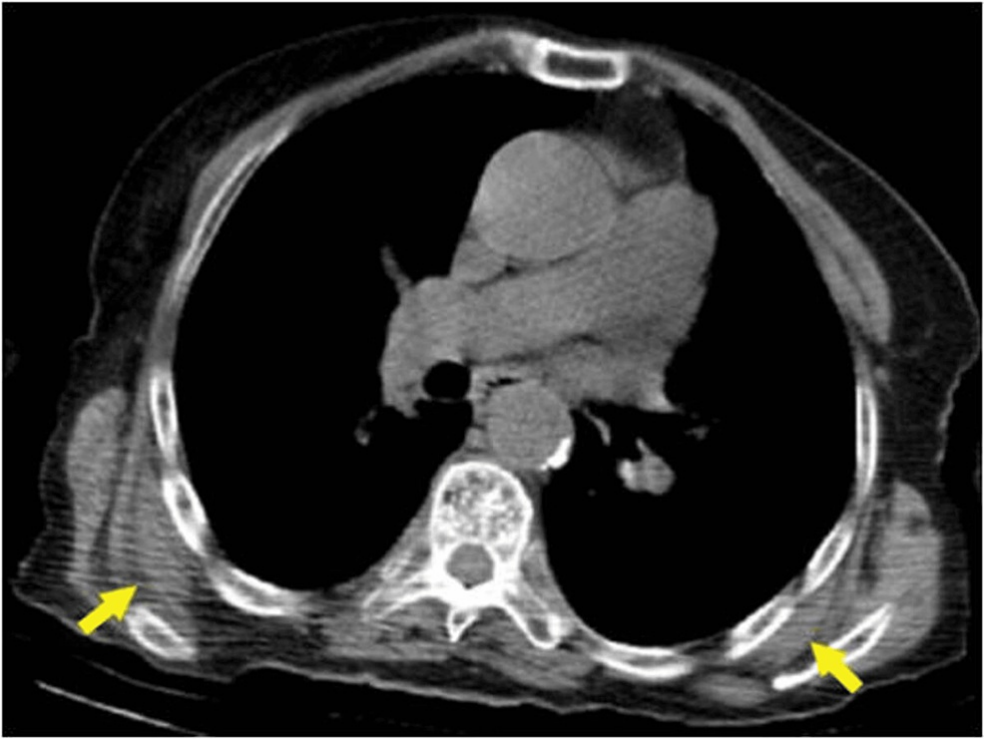
Axial non-contrast CT image of a 85-year-old male patient showing (yellow arrows) bilateral ED; soft tissue located at the posterior thoracic wall, well-circumscribed and isodense with the adjacent muscle structure.

Unilateral ED was detected in 32 (40.5%) of the patients and bilateral ED in 47 (59.5%) (Figure 2). Right-side mass was present in 20 (25.3%) patients, while left-side mass was found in 12 (15.2%) (Table 1 ).

In the cases determined with ED, the median age was 75 years (range, 53-96 years), and ED was not determined in any patient aged <50 years. When the patients aged >50 years were separated into two groups of 50-70 years and 70-99 years, the risk of ED presence was determined to be 27.78-fold greater (95% CI: 8.85-90.91) in patients aged 70-99 years compared to those in the 50-70-year age group.

**Figure 3:**
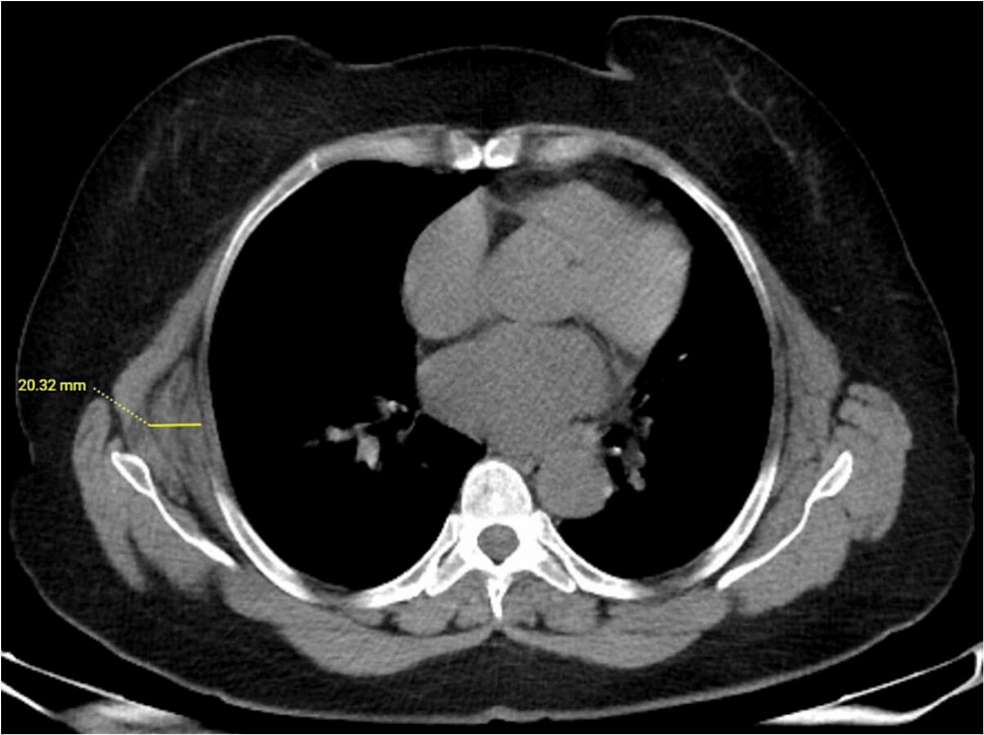
Axial non-contrast CT image of a 65-year-old female patient with right unilateral ED. The thickness measurement of ED was made by measuring the widest diameter on the axial image.

When the ED thickness was evaluated, the median thickness was determined to be 12 mm (range: 7-25 mm) for right-side ED and 11 mm (range: 5-25 mm) for left-side ED (Table 1 and Figure 3).

**Figure 4:**
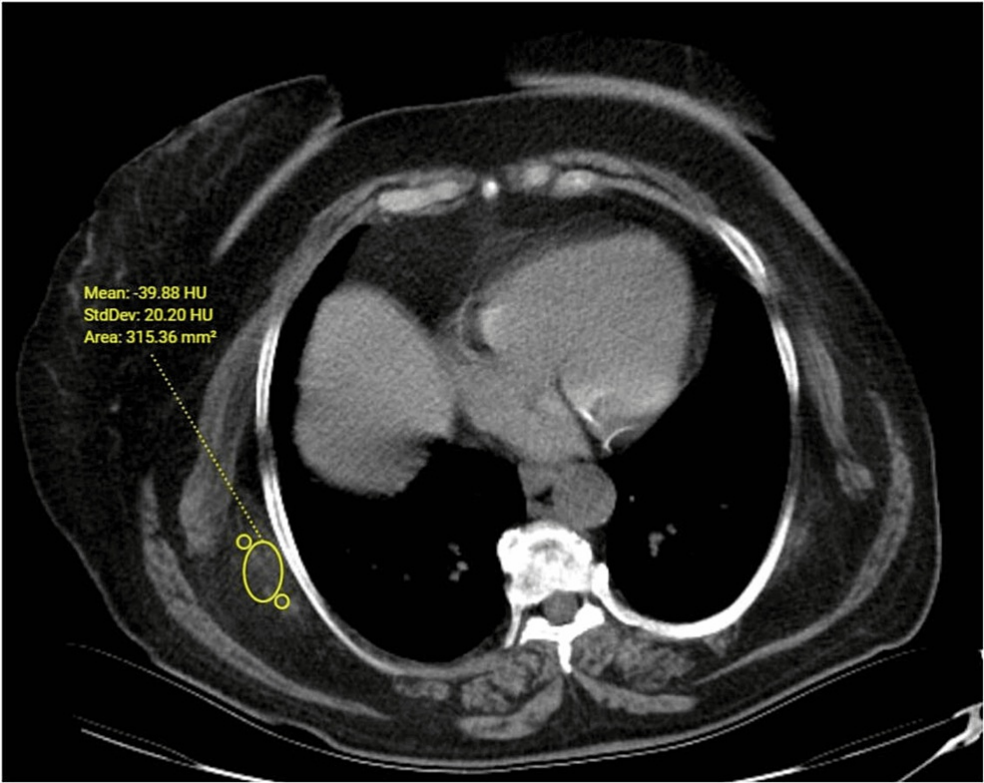
Axial non-contrast CT image. Density was measured to be -39 HU for right-side ED in a 68-year-old female patient.

**Figure 5:**
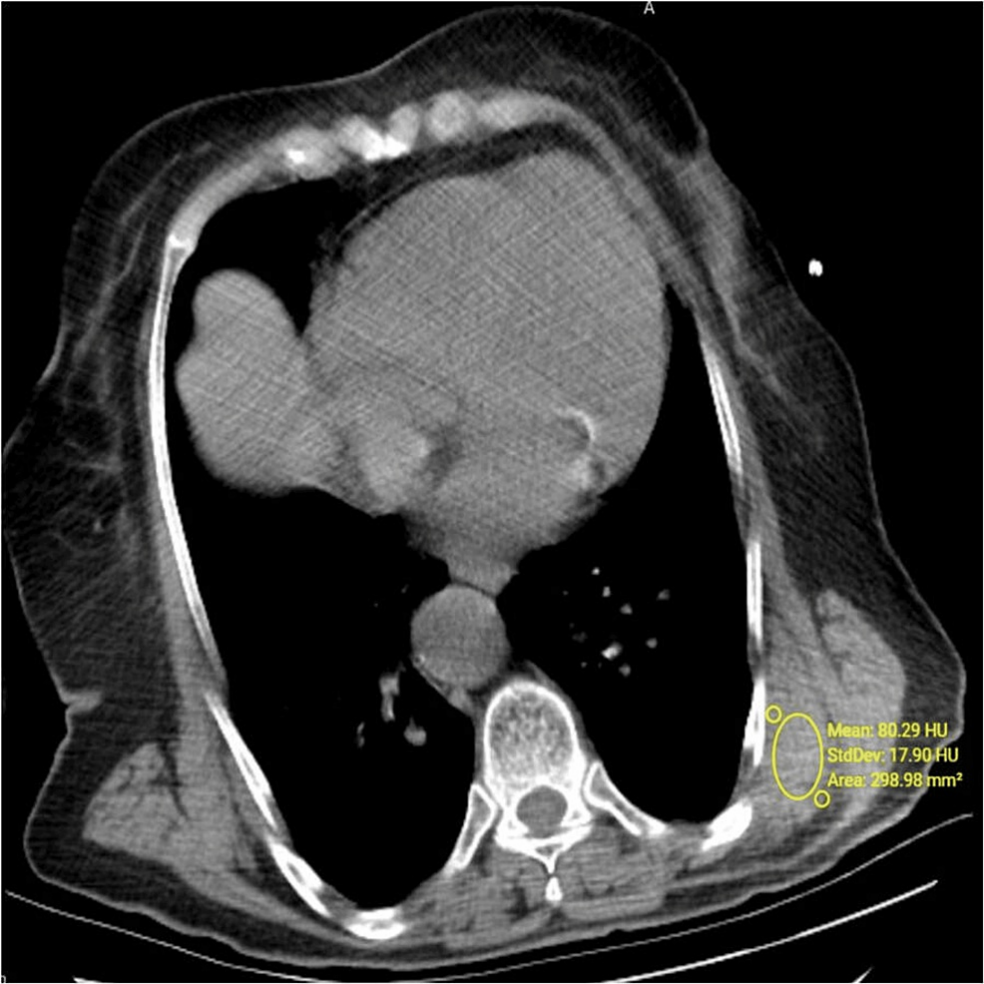
Axial non-contrast CT image. Density was measured to be +80 HU for left-side ED in a 78-year-old female patient.

The median density was determined to be 27 HU (range: -41 to 70 HU) for right-side ED and 25.5 HU (range: -46 to 90 HU) for left-side ED (Table 1 and Figures 4, 5).

A statistically significant correlation was determined between age and the presence of ED (p < 0.001). No statistically significant correlation was found between age and ED thickness or density (p = 0.602, p = 0.233, respectively). No statistically significant correlation was determined between gender and ED thickness, between gender and ED density, or between gender and the side of localization (p = 0.297, p = 0.570, p = 0.456, respectively). The original radiology report of each patient was reviewed. In the 79 patients determined with ED, it was seen that the lesion had not been reported in the first radiological evaluation of any of these patients.

## Discussion

ED is an uncommon, benign, poorly circumscribed connective-tissue tumor classically located in the subscapular region and is four times more common in females [7,8]. Similarly, in the current study, the frequency of ED in females was found to be 3.65-fold greater than in males. It has been stated in the literature that the risk of ED increases with age, with the mean age reported to be 60 years [7,8]. In an autopsy series of 235 cases, Jarvi and Lansimies did not encounter any case of ED under the age of 58 years [9]. The current study results showed a significant increase in ED frequency together with increasing age, especially in the patients aged >70 years, and no case of ED was determined in those aged <50 years. Therefore, if there are typical appearance and location on the CT or magnetic resonance imaging (MRI) examinations of the older age group, the diagnosis of ED is at the forefront, and there is no need for further diagnostic tests. However, in cases aged <50 years or in the absence of typical imaging findings, tumors, such as lipoma, neurofibroma, dermoids, malignant fibrous histiocytoma, and sarcoma, should be considered in the differential diagnosis for soft tissue masses determined in the chest wall and when necessary should be confirmed with histopathological diagnosis [10].

The typically seen localizations of ED are between the scapula and thoracic wall, between the rhomboid major, latissimus dorsi, and serratus anterior muscles in the inferior subscapular region [2,7]. The ED cases in the current study were seen to be in typical localizations, often bilateral and more often on the right side in unilateral cases, consistent with the literature. This supports the theory of mechanical friction in the etiology.

In patients with clinical symptoms, there may be pain in the back and shoulder, soft tissue swelling, and a clicking sound during shoulder movement. Ultrasonography (US) is the first preferred method in the imaging of symptomatic patients as it is non-invasive and easily accessible [7]. On US, ED in typical localizations can be seen to be echogenic with superficial localisation as a mass in a multilayer pattern of linear hypoechoic areas of fat tissue within fibroelastic tissue or as a homogenous hypoechoic mass [11]. However, in daily practice, more than 50% of patients are asymptomatic and are detected incidentally during CT or MRI evaluations [6]. On CT, ED is seen as an inhomogeneous, poorly circumscribed, non-encapsulated soft tissue mass in the subscapular region, containing lower-density linear fatty tissue structures and similar density with adjacent muscle structures. On T1- and T2-weighted MRI images, it is seen as a mass with similar signals to those of muscle tissue and including linear signal changes of fat. Heterogeneous contrast enhancement can be observed with gadolinium [12]. Benign mass characteristics can be observed on diffusion-weighted imaging [6,13].

In the current study, the frequency of ED was determined to be 2.4%. There is a limited number of studies in the literature with extensive patient series. Tepe et al. reported ED frequency of 2.73% in a study of 4,074 cases, which was consistent with the current study [10]. Different prevalence rates, such as 0.8% (aged >50) and 2% (aged >60), have been reported in other studies [9,14]. However, in an autopsy series, prevalence in cases aged >55 years was reported as 24.4% in females and 11.2% in males [15].

Bilateral ED was observed most often in the current study, and of the unilateral cases, localization was more often on the right side than the left. The tendency of ED to be bilateral has been shown in many studies, and it has been reported at rates varying between 12% and 73% [8]. The frequency of ED was found to be 51.4% bilaterally and 48.6% unilaterally (28 on the right, 26 on the left) in the study by Tepe et al. [10]. The frequency of ED was found to be 30.56% bilaterally and 69.4% unilaterally (21 on the right, 4 on the left) in the study by by AlAwaji et al. [14]. Similar to the current study, it was shown in an autopsy series to occur predominantly on the right side, but it was seen bilaterally in up to 50% of cases [15].

Soft tissue masses of ED may be of varying size and thickness. In the current study, we found that the mean ED tissue thickness was similar on both sides. No significant correlation was detected between the average ED tissue thickness and age or gender. This may show that ED thickening is not expected with increasing age. The size of ED mass was also reported not to be linked to age or gender in the study by Alawaji et al. [14].

To the best of our knowledge, no previous study has been conducted with the ED density measurement. In the current study, the tissue density of ED was measured, and a wide density range of -40 HU to +90 HU was seen. These density values are related to the varying degrees of fibrous and fatty structures that the ED comprises. The internal nature of a heterogenous solitary soft tissue mass can be evaluated in more detail on MRI. Although it has been stated that biopsy is often unnecessary for diagnosis, it is recommended in suspicious cases with atypical clinical and radiological findings without a contralateral mass [2].

Fine needle biopsy is not recommended because of the hypocellular nature of ED [16]. Conservative follow-up is sufficient in the treatment of asymptomatic masses and lesions smaller than 5 cm, while marginal removal is necessary in symptomatic cases [17].

A striking finding of this study was that the EDs determined on CT imaging were not mentioned in the first radiological reports of any of these cases. In the prevalence study in elderly patients conducted by Naylor et al., in a series of 12 patients with 21 elastofibromas, only four of 21 tumors were mentioned in initial radiology reports [12]. In another study similar to our study, no case of ED was mentioned in the initial reports [9]. Although diagnosis can be made easily on CT, the reason that this often were overlooked was thought to be due to the ED appearance mimicking the serratus anterior muscle fibers and that the density was similar to that of the adjacent muscle and fat tissue.

This study had some limitations, primarily that the number of patients and single-center design could be considered insufficient for a prevalence study. Because one goal of the study was to measure lesion density, only non-contrast CT examinations were considered, and hence it was not possible to determine the contrast pattern of the ED lesions. Thickness measurements taken from axial slices as lesion size and three-dimensional and volume measurement with reformatted images may have provided a more accurate result. Another limitation was that there was no advanced MRI examination because of the retrospective design and that the ED cases were not stated in the first radiology reports; hence, there could be no confirmation with pathological diagnosis. The other limitation was the inability to analyze interobserver and intra-observer variability, because the CT images were examined by a single radiologist.

## Conclusions

ED is a soft tissue mass that is mostly detected incidentally and mainly affects people over the age of 50 years and predominantly females. Although pathological diagnosis is not usually required because of pathognomonic CT and MRI findings, it can be easily overlooked on cross-sectional examinations because of the similar appearance and density to adjacent structures. Knowledge of the characteristic radiological features of these lesions and increased awareness of radiologists will make early diagnosis possible in asymptomatic cases.
